# Expression of TRPC6 in Renal Cortex and Hippocampus of Mouse during Postnatal Development

**DOI:** 10.1371/journal.pone.0038503

**Published:** 2012-06-06

**Authors:** Pengjuan Xu, Jing Xu, Zhigui Li, Zhuo Yang

**Affiliations:** College of Medicine, Nankai University, Tianjin, China; The University of Manchester, United Kingdom

## Abstract

TRPC6, a member of the TRPC family, attracts much attention from the public because of its relationship with the disease. In both the brain and kidney, TRPC6 serves a variety of functions. The aim of the present study was to observe the expression and effects of TRPC6 in renal cortex and hippocampus during early postnatal development of the mouse. In the present study, immunohistochemistry and Western blotting were used to detect the expression of TRPC6 in the mouse kidney and hippocampus of postnatal day 1, 3, 5, 7, 14, 21, 28 and 49 (P1, P3, P5, P7, P14, P21, P28 and P49). Results showed that the expression of TRPC6 was increased in the mouse hippocampus, and there was a significant increase between P7 and P14 during the postnatal development. Meanwhile, the expression of TRPC6 was also detected in glomerulus and tubules, and a decreased expression was found during postnatal maturation of mouse renal cortex. From these *in vivo* experiments, we concluded that the expression of TRPC6 was active in the developing mouse kidney cortex, and followed a loss of expression with the development of kidney. Meanwhile, an increased expression was found in the hippocampus with the development. Together, these data suggested that the developmental changes in TRPC6 expression might be required for proper postnatal kidney cortex development, and played a critical role in the hippocampus during development, which formed the basis for understanding the nephrogenesis and neurogenesis in mice and provided a practically useful knowledge to the clinical and related research.

## Introduction

Transient receptor potential channels (TRPCs), which mediate calcium, sodium and magnesium ion influx, are non-selective cation channels associated with six-transmembrane domains in membrane [1], [2]. As the sensor to changes in cellular local environments, TRPCs have diverse functions including mechanical and taste sensing [3] as well as maintaining ion homeostasis. Recently, TRPCs have drawn more and more attention because mutated TRPCs are connected to the pathophysiology and some specific diseases [4], such as asthma [5], idiopathic pulmonary arterial hypertension (IPAH) [6], chronic obstructive pulmonary disease (COPD) [7] and so on.

TRPCs genes encode subunits that form ion channels in many types of cells [8], [9], [10]. Members of the TRPC family have been identified on the basis of amino acid sequence and are classified into four families [11]. TRPC6, a member of the TRPC family, contains seven members separated by four subfamilies of channel subunits that serve a variety of cellular functions [11], [12]. In the kidney, TRPC6 is enriched in the podocyte foot processes and the collecting ducts. It functions as a critical regulator of the normal renal function. Associated with slit diaphragm proteins-nephrin and podocin, TRPC6 composes the slit diaphragm complex [8]. The mutant TRPC6 may affect the functions of this complex, leading to abnormalities in podocyte foot processes of many renal diseases [13], [14], [15]. There are various studies suggesting that mutant TRPC6 is closely correlated with the mechanism of hereditary and non-hereditary nephropathies, such as focal and segmental glomerulo sclerosis (FSGS) [16], [17], [18], minimal-change disease (MCD) and membranous glomerulonephritis (MN) [19].

On the basis of previous findings, the TRPC6 expression level and channel function may contribute to the pathogenesis of kidney disease via a dysregulated Ca^2+^ influx [19].

Moreover, TRPCs are widespread in many tissues including the central nervous system (CNS), and play as important regulators during the development [20], [21]. Some studies reported that TRPC6 was mainly localized to proximal dendrites and the axon hillock,and involved in the brain-derived neurotrophic factor (BDNF)-mediated growth cone tuning, neuron survival and spine formation [20], [22], [23].

Mossy fiber synapse remodeling and the down regulation of TRPC6 expression were closely related. TRPC6 promoted dendritic growth via the Ca^2+^/camodulin-dependent protein kinases IV and cAMP-response-element binding protein (CaMKIV-CREB)-dependent pathway, and played a critical role in dendritic growth during the early development, especially at the stages when neuronal activity was low [24].

Recently, many studies have focused on effects of gain-of-function mutations in the TRPC6 gene. However, little is known about the postnatal development in density and properties of TRPC6 channels. Moreover, strong indications of the involvement of channels in several functions come from relations to the level of channel expression. Therefore, in the present study, we examined and compared the expression and effects of TRPC6 in the renal cortex and hippocampus during mouse postnatal development using immunohistochemistry and Western blotting methods.

## Results

### 1. Immunohistochemical Detection of TRPC6 in the Mouse Renal Cortex

**Figure 1:**
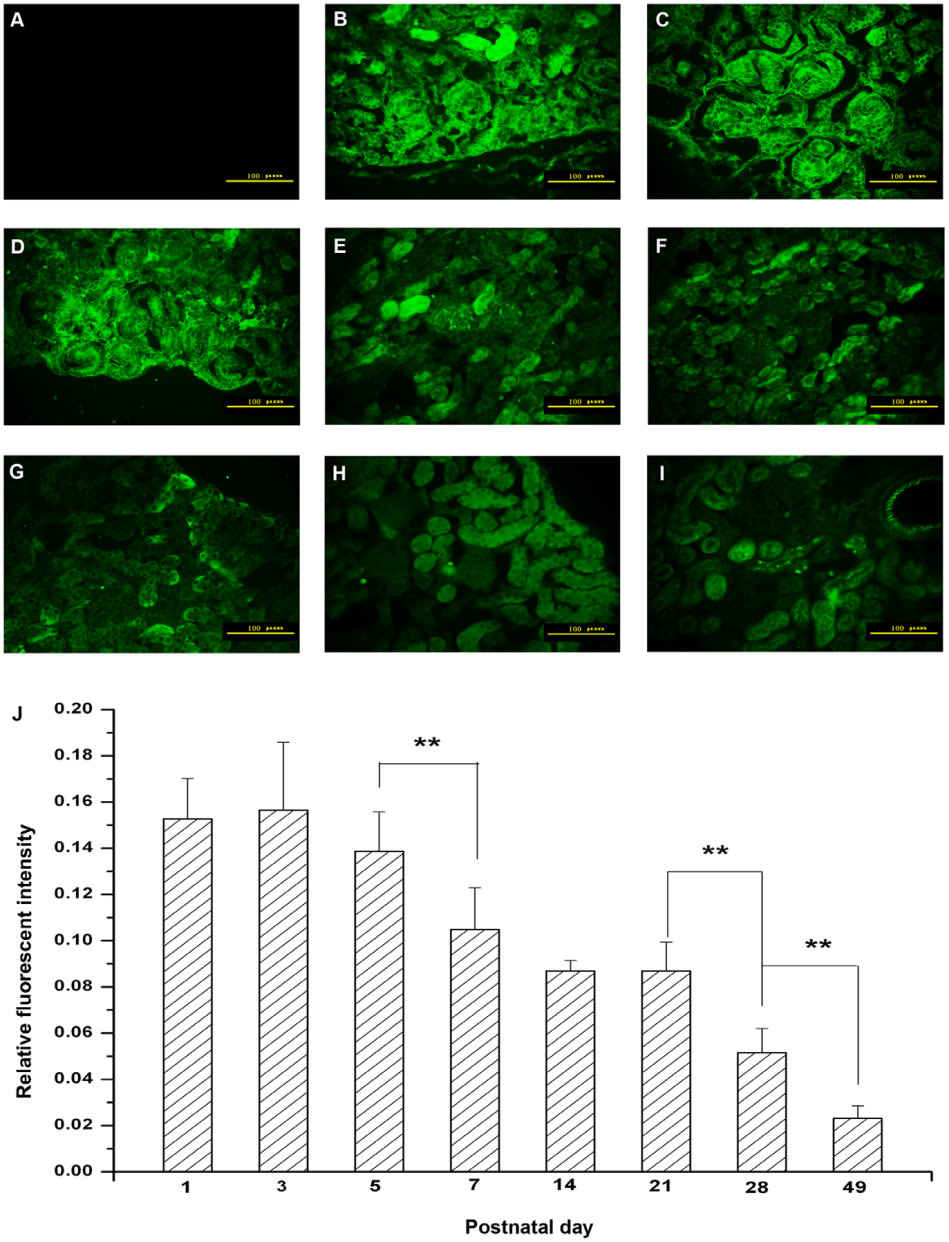
Localization of TRPC6 in mouse renal cortex during postnatal development. Immunohistochemical staining of TRPC6 showed the expression of TRPC6 in the mouse renal cortex at P1 (B), P3 (C), P5 (D), P7 (E), P14 (F), P21 (G), P28 (H), and P49 (I). The negative control image was from the renal cortex, in which the primary antibody was a species-appropriate IgG (A). The corresponding linear diagram of relative fluorescent intensity in glomeruli was shown in (J). The sections from P1 to P5 showed that TRPC6 mostly expressed in comma-shaped body, S-shaped body and renal corpuscles of stage III (B-D). After P7 the expression was found in renal corpuscles and it was weakly positive (E-F). During the development, tubules were all observed. Scale bar, 100 µm. Data were presented as means±S.D. n = 6/group. *P<0.05, **P<0.01 *vs.* adjacent age group.

To identify the morphological and developmental changes in the protein expression, the specific antibody was used to mark the TRPC6 detection during the postnatal development in mice. It was known that there were four developmental stages of renal corpuscles: comma-shaped body, S-shaped body, renal corpuscles of stage III and renal corpuscles of stage IV [25]. In the renal cortex of immature mouse postnatal day 1–5 (P1–P5), it was observed that S-shaped body appeared mostly in every stage of renal corpuscles and comma-shaped body (Figure 1. B-D). At P7, there were renal corpuscles of stage IV only (Figure 1. E). Moreover,the number of corpuscles was proliferated prominently from P1 to P7. And the volume of cortex was increased after P7.

TRPC6 was found in all stages of renal corpuscles and tubules. Moreover, the expression of TRPC6 in glomeruli was decreased significantly during the postnatal development (Figure 1. J).

### 2. Western Blotting Detection of TRPC6 in the Mouse Renal Cortex

**Figure 2:**
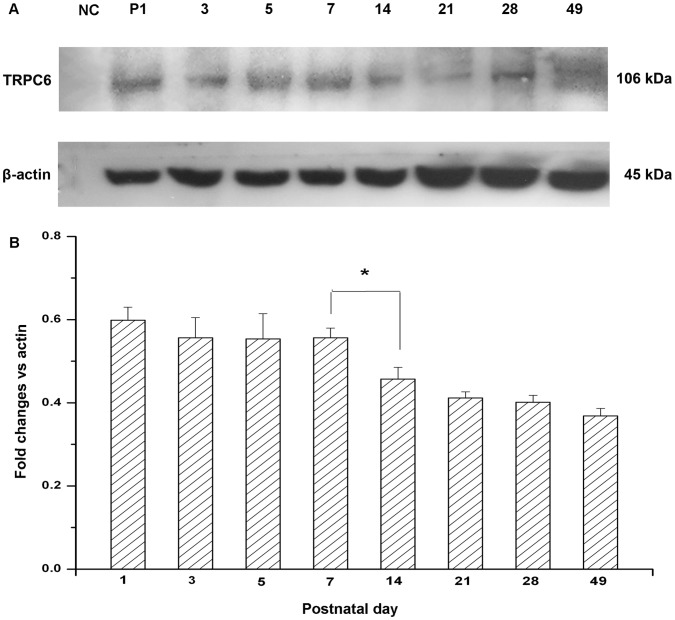
Immunoblot analysis of TRPC6 in mouse renal cortex during the development. The position of 106 kDa molecular size was the expression pattern of TRPC6 in renal cortex at the indicated ages (A). Quantitative determination of immunoblot showed significant changes in TRPC6 expression in mouse renal cortex during the development (B). The expression of TRPC6 was normalized to β-actin expression. Data were presented as means±S.D. *P<0.05, **P<0.01 *vs.* adjacent age group.

To identify developmental changes in the protein expression, the expression of TRPC6 was examined by Western blotting as β-actin was a consult. For these experiments, the cortex was separated from the kidney. As seen in Figure 2. A, single immunoreactive bands of 106 kDa were observed from cortex lysates. In addition, the quantitative immunoblot analysis showed that the expression of TRPC6 was decreased. During this period, the biggest change of TRPC6 level was between P7 and P14, and it was stable after P28 (Figure 2. B).

### 3. Immunohistochemical Detection of TRPC6 in the Mouse Hippocampus

**Figure 3:**
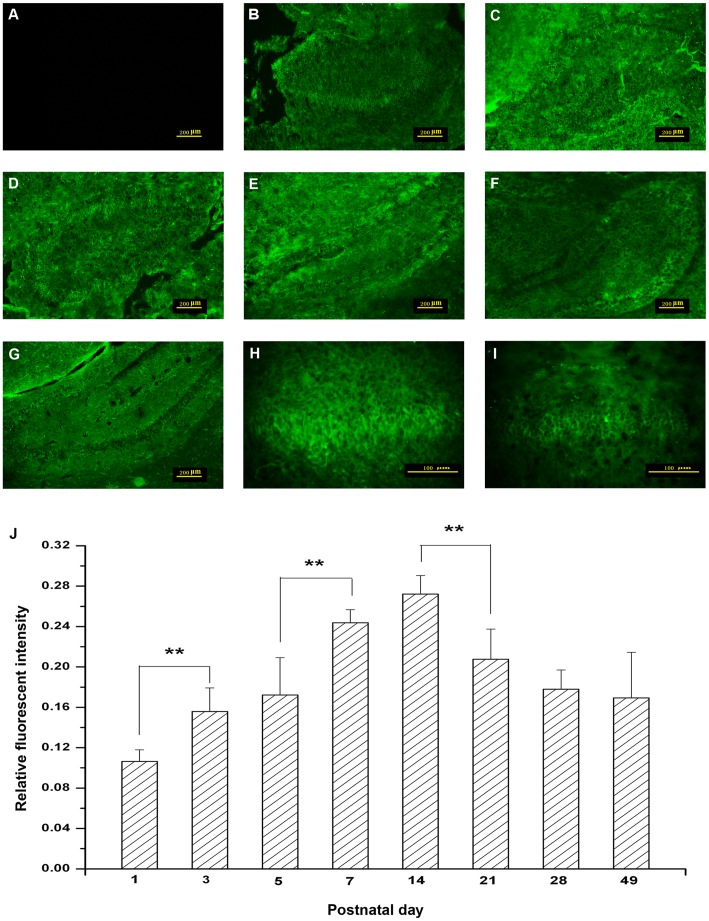
Localization of TRPC6 in mouse hippocampus during the postnatal development. The immunohistochemical staining showed that TRPC6 was expressed in all regions of the hippocampus at P1 (B), P3 (C), P5 (D), P7 (E), P14 (F) and P21 (G). The negative control image was shown in (A). Scale bar, 200 µm. In suit hybridization of P1 (H) and P5 (I) brain sections showed the expression of TRPC6 in hippocampal neurons. Scale bar, 100 µm. The corresponding linear diagram of relative fluorescent intensity in glomeruli was shown in (J). Data were presented as means±S.D. n = 6/group. *P<0.05, **P<0.01 *vs.* adjacent age group.

It was found that the hippocampus developed and maturated gradually after birth. The volume of hippocampus increased slowly before P7 (Figure 3. B-E), and turned to be fast from P7, with a peak at P14 (Figure 3. F). After this fluctuation, the development rate became stabilize (Figure 3. G).

As a result, it was also found that TRPC6 was expressed in all regions of the mouse hippocampus during the postnatal development. Moreover, the expression of TRPC6 was increased significantly in hippocampus during the postnatal development (Figure 3. J).

### 4. Western Blotting Detection of TRPC6 in the Mouse Hippocampus

**Figure 4:**
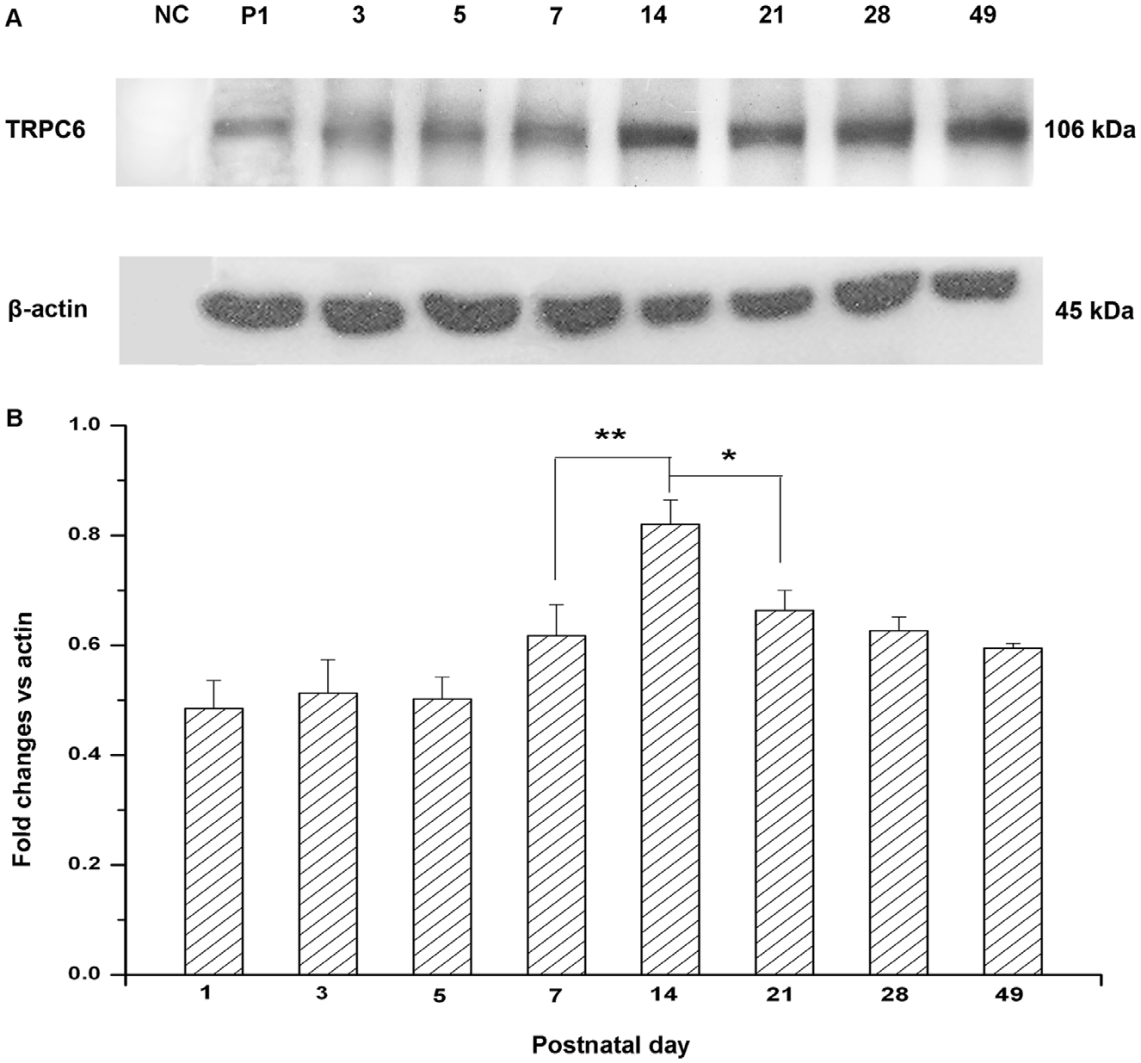
The immunoblot analysis of TRPC6 in mouse hippocampus during the development. Total lysates of tissues treated as indicated blotted with antibodies to TRPC6 at the indicated ages (A). Quantitative determination of TRPC6 expression showed an abrupt increase between P7 and P14 in mouse renal cortex during the development, and expression levels remained higher into adulthood (B). The expression of TRPC6 was normalized to β-actin expression. Data were presented as means±S.D. *P<0.05, **P<0.01 *vs.* adjacent age group.

As shown in Figure 4, the expression of TRPC6 channels was increased in hippocampus during the development with the expression of β-actin for normalization. There was an abrupt increase between P7 and P14 (Figure 4. B). The expression levels remained higher into adulthood. Before P7 and P14, the expression of TRPC6 also increased but at the slower rate. In the mouse hippocampus,the expression of TRPC6 channels was higher than that in the renal cortex.

## Discussion

The TRPCs were proposed to act as Ca^2+^-permeable cation channels that were activated in response to stimulation of G protein-coupled receptors [26], [27], [28], which were expressed in a variety of multicellular organisms with different functions [12]. Moreover they can be divided into four subfamilies by homology and function: TRPC1, TRPC2, TRPC4/5 and TRPC3/6/7 [11].

The previous study showed that TRPC6 was a calcium-permeable cation channel that can be directly activated by diacylglycerol (DAG) [29]. TRPC6 was expressed in many different regions and organs, such as hippocampus [20], substantia nigra [23], lung, epididymis, skeletal muscle [30], kidney [31] and so on. In the brain, TRPC6 expression was found on interneurons, Purkinje cells and post-mitotic granule cells. The down-regulation of TRPC6 might be required during the postnatal development of cerebella neurons for initiation of differentiation and migration of interneurons [32]. Recent studies showed that TRPC6 revealed an influence on the CNS [22] and was important for the increase in intra-cellular Ca^2+^ following combining of G-protein-coupled receptors and receptor tyrosine kinases, which was critical for synaptic plasticity and memory [33], [34].

In the kidney, TRPC6 was co-localized with the human disease-associated slit diaphragm proteins nephrin and CD2-associated protein (CD2AP) [8], [14], [15], [35]. Moreover, large-conductance calcium-activated potassium (BK_Ca_) channels were shown to interact directly with Ca^2+^-permeable TRPC6 channels in podocytes [36]. Mutations in TRPC6 affected the calcium channel function and caused many diseases [8], [16], [17]. As found in this study there was a continuous change during the postnatal development in the kidney and in the hippocampus. The question is whether TRPC6 channels play an important role in hippocampus and renal cortex during the postnatal development.

The immunolocalization with fluorescence and Western blotting was used to determine the expression of TRPC6 in mouse hippocampus. Results showed that there was continuous TRPC6 expression throughout the mouse brain development (Figure 3). Moreover, the peak of TRPC6 expression was found between P7–P14, which was in agreement with the previous study [37]. That period was known to be important for the synaptogenesis *in vivo* [38]. The hippocampus plays an important role in learning, memory and recognition of novelty. Zhou *et al* showed that TRPC6 was mainly localized in excitatory postsynaptic sites in the hippocampus, which indicated that TRPC6 channels acted as an important mediator for sensing extracellular signals that affected the synaptic and behavioral plasticity.

In the kidney, Winn *et al* first found that TRPC6 was specific staining within the glomerulus and the epithelium of surrounding tubules in the normal human renal cortex using the immunofluorescence [39]. Reiser *et al* showed that TRPC6 was expressed widely in glomerulus and tubules, and in glomerulus,TRPC6 was mainly localized in the cell body of podocytes and in primary processes. TRPC6 expression was also detected in glomerular endothelial cells and a few mesangial cells [8]. Moller *et al* found that TRPC6 staining was appeared in glomeruli, especially predominantly within the capillary loop [19]. In the present research, the expression of TRPC6 was found in glomeruli and tubule, and the expression of TRPC6 was decreased during the postnatal development.

As we know, in mammals and birds, there are three stages in the kidney development including pronephros, mesonephros and metanephros, but only the metanephros differentiates into the permanent kidney [40]. It is noteworthy that interactions of cellular protein, signaling molecules and pathways seem to be involved in the development of kidney. Some researches indicated that the subcapsular zone was disappeared after birth, and there was no glomerulus producing. From P7 to adulthood, the developing characteristic of glomerulus was marked by the volume growth of average unit. We observed that there was continuous TRPC6 expression throughout the development of mouse kidney cortex (Figure 1) and the expression of TRPC6 was decreased between P7 and P14. Our present findings indicated that TRPC6 might play an important function at the beginning of the kidney development and play a critical role in the mature kidney.

In conclusion, TRPC6 was expressed in all regions of the hippocampus in various levels during the development, and may promote the formation of excitatory synapses in hippocampus [24]. In the kidney cortex, TRPC6 was expressed in the glomeruli and tubule, and it could be detected in all stages during the glomeruli development. All these findings revealed a novel annotation of TRPC6 during the development of the CNS and kidney, which provided a new handhold for us to understand the development of hippocampus and kidney, as well as the mechanism of learning or memory and kidney diseases in the future.

## Materials and Methods

### 1. Ethics Statement

The animal experiments were performed in accordance with institutional guidelines, and the study was approved by the ethics committee of Nankai University. Experiments were designed to minimize the number of animals used and their suffering.

### 2. Antibodies and Reagents

Rabbit polyclonal anti-TRPC6 antibodies (primary antibody, working dilution 1∶1000, Abcam, Cambridge, MA, U.S.A); Alexa 488- conjugated goat anti-rabbit IgG antibodies(secondary antibody, working dilution 1∶1000, Invitrogen, San Diego, CA, U.S.A); rabbit polyclonal anti-β-actin IgG (primary antibody, working dilution 1∶1000, Santa Cruz Biotechnology, Inc. CA, U.S.A); chemiluminescent HRP substrate (Immobilon western, Millipore Corporation, Billerica, MA, U.S.A).

### 3. Animals

All animal procedures were accorded to protocols approved by the Animal Care Committee of the Animal Center at the Chinese Academy of Sciences in Shanghai. Adult C57/BL6 mice (20 g, 8–10 weeks old) were purchased from the Experimental Animal Center of the Chinese Academy Medical Sciences. Animals were maintained under standard laboratory conditions under artificial 12 hours light/12 hours dark cycle. Two females were paired with one male (2∶1) for a period of 4–5 days until mating. The day of birth was recorded as P0. The brain and kidney were collected on P1, P3, P5, P7, P14, P21, P28 and P49.

### 4. Immunohistochemistry

Tissue samples were placed in 4% paraformaldehyde in phosphate-buffered saline (PBS) over 2 hours at 4°C, immersed in 30% sucrose overnight at 4°C, and then were embedded in OCT compound (Tissue-Tek, Miles) and sectioned at 5 µm (Leica CM 1850, Leica Instruments) for the morphological and immunohistochemistry study. Sections were washed three times in PBS for five minutes each and incubated in blocking buffer with 10% serum of the secondary antibody host species for 1 hour at room temperature. After that, sections were incubated with primary antibodies including anti-TRPC6 overnight at 4°C. After being washed three times for 10 minutes with PBS at room temperature, sections were incubated with Alexa 488-conjugated anti-rabbit IgG for 3 hours at room temperature. Negative control samples were treated with species-appropriate IgG instead of primary antibody. The fluorescent signals were examined using a Leica TCS SP5 laser-scanning confocal microscope. For analyze of TRPC6 expression, fluorescent intensity was quantified by measuring intensity in tissues using Image-Pro Plus. Data were analyzed from three sections of one sample, and there were six samples for each age group. The TIFF images were not processed before measurement of signal intensities.

### 5. Western Blotting

Kidneys and brains were isolated, and the cortex and hippocampus were isolated by the dissection. Then the cortex and hippocampus were minced into pieces and lysed in lysis buffer containing a proteinase inhibitor cocktail (1∶100 dilutions). Lysates were centrifugated at 12000 r/min for 7 minutes at 4°C. The supernatant was collected and total proteins were quantified by bicinchonic acid assay according to manufacturer’s instructions. Protein samples were fractionated by SDS-PAGE and were electrotransferred to polyvinylidene difluoride (nitrocellulose) membranes in Tris-glycine buffer at 300 mA for 1.5 hours. Then membranes were incubated with blocking buffer which was 5% fat-free milk for 2 hours, followed by incubation with primary antibody described above (anti-TRPC6) for overnight at 4°C. After that, membranes were washed three times with Tris-buffered saline/Tween 20 (TBST) buffer and incubated with a 1∶2500 dilution of horseradish peroxidase-conjugated secondary antibodies for 1 hour at room temperature. Blots were washed another three times with TBST and detected with chemiluminescent HRP substrate (Immobilon western). Negative control samples were treated with species-appropriate IgG instead of primary antibody. The figures showed representative results from experiments repeated at least three times. For the analysis, quantitation was performed by scanning and determination of the intensity of the hybridization signals. Image J was used to evaluate differences between the sample of interest and its respective β-actin.

### 6. Statistics

All data were expressed as mean±standard deviation (S.D.) and analyzed by Origin 8.0 and SPSS 17.0. There was a minimum of six animals per age group. Results were analyzed by a one-way ANOVA followed by the Newman-Keuls post-hoc test, and *P<*0.05 was considered significant.
